# Genomic insights into differentiation and adaptation of *Amorphophallus yunnanensis* in the mountainous region of Southwest China

**DOI:** 10.1002/ece3.10861

**Published:** 2024-01-23

**Authors:** Yong Gao, Dongqin Dai, Haibo Wang, Weijia Wu, Penghui Xiao, Lifang Wu, Xiaomei Wei, Si Yin

**Affiliations:** ^1^ College of Biological Resource and Food Engineering Qujing Normal University Qujing Yunnan China

**Keywords:** *Amorphophallus yunnanensis*, environmental adaptation, genetic differentiation, geographical isolation, mountainous region of Southwest China

## Abstract

The role of geographical isolation and environmental adaptation in driving the differentiation and radiation of species has been a hotspot in evolutionary biology. The extremely complicated and fragmented geography of the mountainous region of Southwest China provides an excellent system for investigating the process of species divergence in heterogeneous habitats. *Amorphophallus yunnanensis* is a species of extreme habitat preference that resides mainly in the mountainous region of Southwest China. Here, we used restriction site‐associated DNA sequencing (RAD‐seq) to characterize the geographic pattern of genetic variation among 19 populations of *A. yunnanensis* as well as the genomic basis of environmental adaptation. A pattern of low population genetic diversity and high level of genetic differentiation was observed. The genomic data revealed a clear east–west genetic differentiation, with two distinct genetic lineages corresponding to the Guizhou plateau and Yunnan plateau, respectively. However, we discovered demographic expansion of the Guizhou Plateau lineage and recent hybridization in populations at the contact region. Significant levels of isolation by distance along with isolation by environment were detected. Outlier tests and genome–environment association analyses identified 89 putatively adaptive loci that might play a role in environmental adaptation. Our results suggest that the genetic divergence of *A. yunnanensis* is attributed to geographical isolation together with divergent selection in the mountainous region of Southwest China.

## Graphical abstract

RAD‐seq was used to characterize the geographic pattern of genetic variation among 19 populations of *Amorphophallus yunnanensis* in the mountainous region of Southwest China. A clear east–west genetic differentiation was revealed, with two distinct genetic lineages corresponding to Guizhou plateau and Yunnan plateau, respectively. Our results suggest that genetic divergence of *A. yunnanensis* is attributed to geographical isolation together with divergent selection.

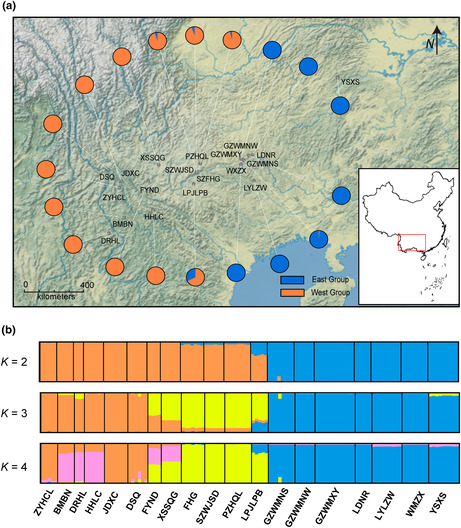

## 1 INTRODUCTION

The genetic divergence of species in heterogeneously isolated habitats has long been a hotspot in evolution studies (Hu et al., 2022; Wang et al., 2019). For species in terrestrially isolated habitats with limited distribution abilities, such as plants, migration and gene flow among populations are restricted to narrow regions, and therefore geographic isolation patterns similar to islands are expected (Gao et al., 2015; Han et al., 2017). For example, a high level of genetic divergence as well as low gene flow among populations has been observed in plant species endemic to terrestrial island‐like habitats (Vasquez et al., 2016). Besides, other studies propose that local adaption may contribute to the allopatric divergence of species in terrestrially isolated habitats (Guo et al., 2016).

The mountainous region of Southwest China is known for its complicated geography causing extreme spatial isolation of resident organisms (Wambulwa et al., 2022; Wang et al., 2013). Due to the diverse topography and ecology, this region has long been regarded as a global hotspot of biodiversity (López‐Pujol et al., 2011). These characteristics make Southwest China an excellent system for studying processes that drive the geographical distribution of biodiversity (Liu et al., 2021). One important question is whether genetic differentiation is driven by complete geographical isolation or by environmental selection. Some researchers claim that fragmented habitats facilitated population isolation and allopatric diversification (Kai & Jiang, 2014). Hu et al. (2022) found that both the complex topography and local selection were major factors causing species divergence in the mountainous region of Southwest China. Nonetheless, more studies are needed to uncover mechanisms underlying the genetic differentiation patterns of the flora in Southwest China.

**FIGURE 1:**
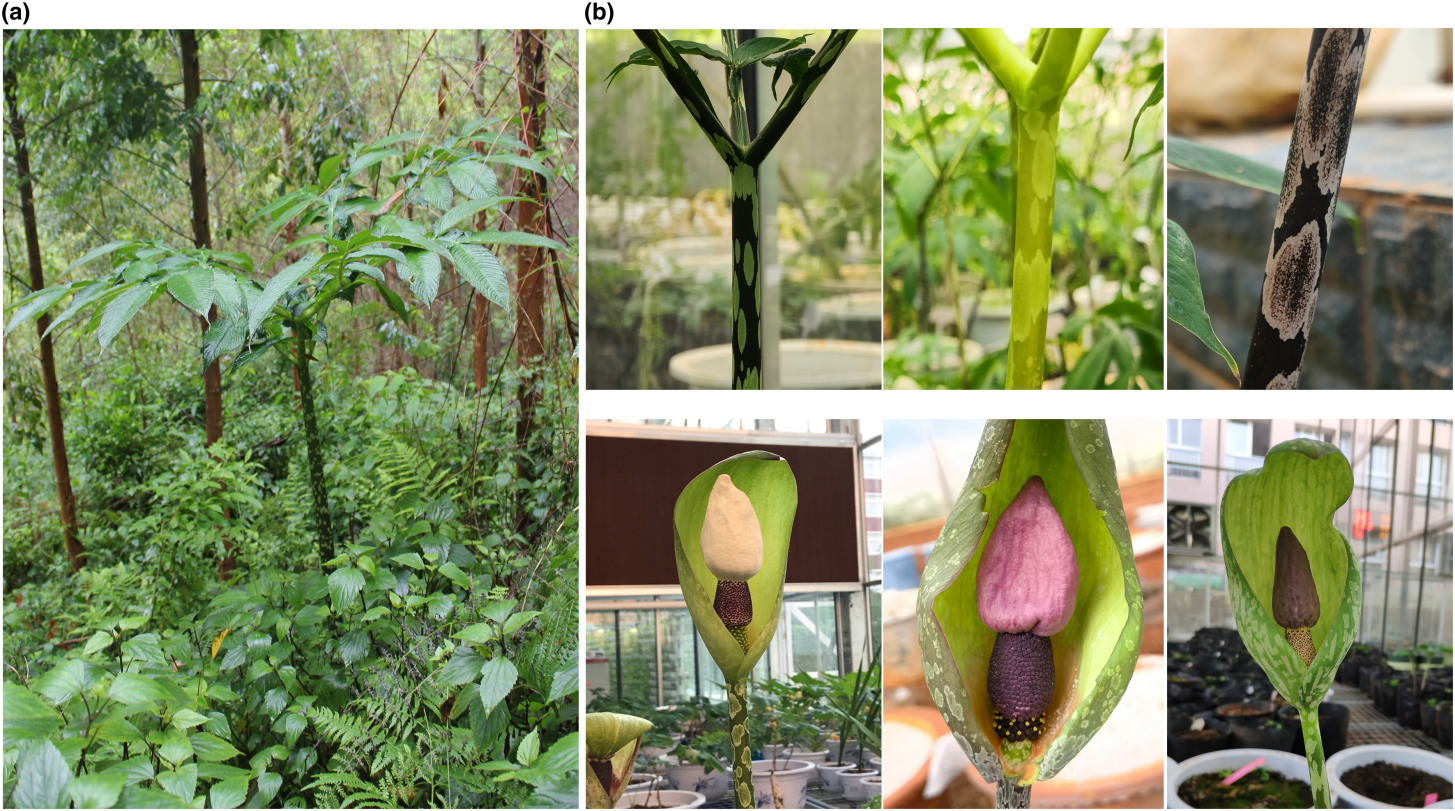
Morphological characteristics of *Amorphophallus yunnanensis*. (a) The plant of *A. yunnanensis*; (b) Variations in the petioles and flowers.

The genus *Amorphophallus* Bl. (Araceae) contains around 219 perennial species distributed across the tropical and subtropical regions of the Palaeotropics from West Africa to Pacific islands (Mayo et al., 1997). Southwest China harbors 17 *Amorphophallus* species, eight species of which are endemic (Li & Hetterscheid, 2010). *A. yunnanensis* is a species located in the mountain ranges of Southwest China and the northern regions of Laos, Thailand, and Vietnam. This species prefers shaded and humid locations with altitudes ranging from 200 to 3300 m, such as primary or secondary forests, thickets, and forest margins (Li & Hetterscheid, 2010). The plant of *A. yunnanensis* is characterized by a solitary leaf and an underground stem, and massive morphological variations have been observed across individuals. The petiole varies from medium to dark olive‐green or dark olive‐brown with rhombic or narrowly elliptic spots in whitish or greenish color (Li & Hetterscheid, 2010). In addition, the appendix of the flower also shows diversifications with several colors observed (Figure 1). Due to its high sensitivity to sunlight and temperature, *A. yunnanensis* is scattered throughout Southwest China in small, isolated populations, making this species an ideal model for inferring the effect of geographic isolation on genetic divergence.

Development in sequencing technologies has created an opportunity to uncover genomic variations from nonmodel organisms. Recently, the first genome assembly in the *Amorphophallu*s genus (*A. konjac*) has been reported, which provides valuable genomic resources for inferring the evolutionary and adaptation mechanisms of *Amorphophallu*s (Gao et al., 2022). However, species in the *Amorphophallus* genus have large genomes (3.70–15.48 Gb), so reduced‐representation library sequencing is a more effective technology for such taxa than whole‐genome sequencing (Gargiulo et al., 2021; Tao et al., 2021). Restriction‐site‐associated DNA sequencing (RAD‐seq) has been employed in population genetic studies of several *Amorphophallu*s species, such as *A. paeoniifolius* and *A. albus* (Gao et al., 2017, 2021). While previous research focuses primarily on the genomic variation of cultivated resources of *Amorphophallu*s, the evolutionary history of wild populations has not been investigated.

The previous study based on SSR markers described the genetic diversity and population structure of *A. yunnanensis* (Yin et al., 2021). However, merely several SSRs lacked the power to infer fine‐scale genetic patterns and functional genomic adaptation. Here, we used RAD‐seq to explore the roles of diverse topography and ecology in causing the genetic differentiation of *A. yunnanensis* in Southwest China. In this study, we (1) draw the genetic variation pattern of this species, (2) infer the demographic history, and (3) investigate the effect of topographic heterogeneity and ecological variation in shaping population differentiation of *A. yunnanensis*.

## 2 MATERIALS AND METHODS

### 2.1 Sample collection

**FIGURE 2:**
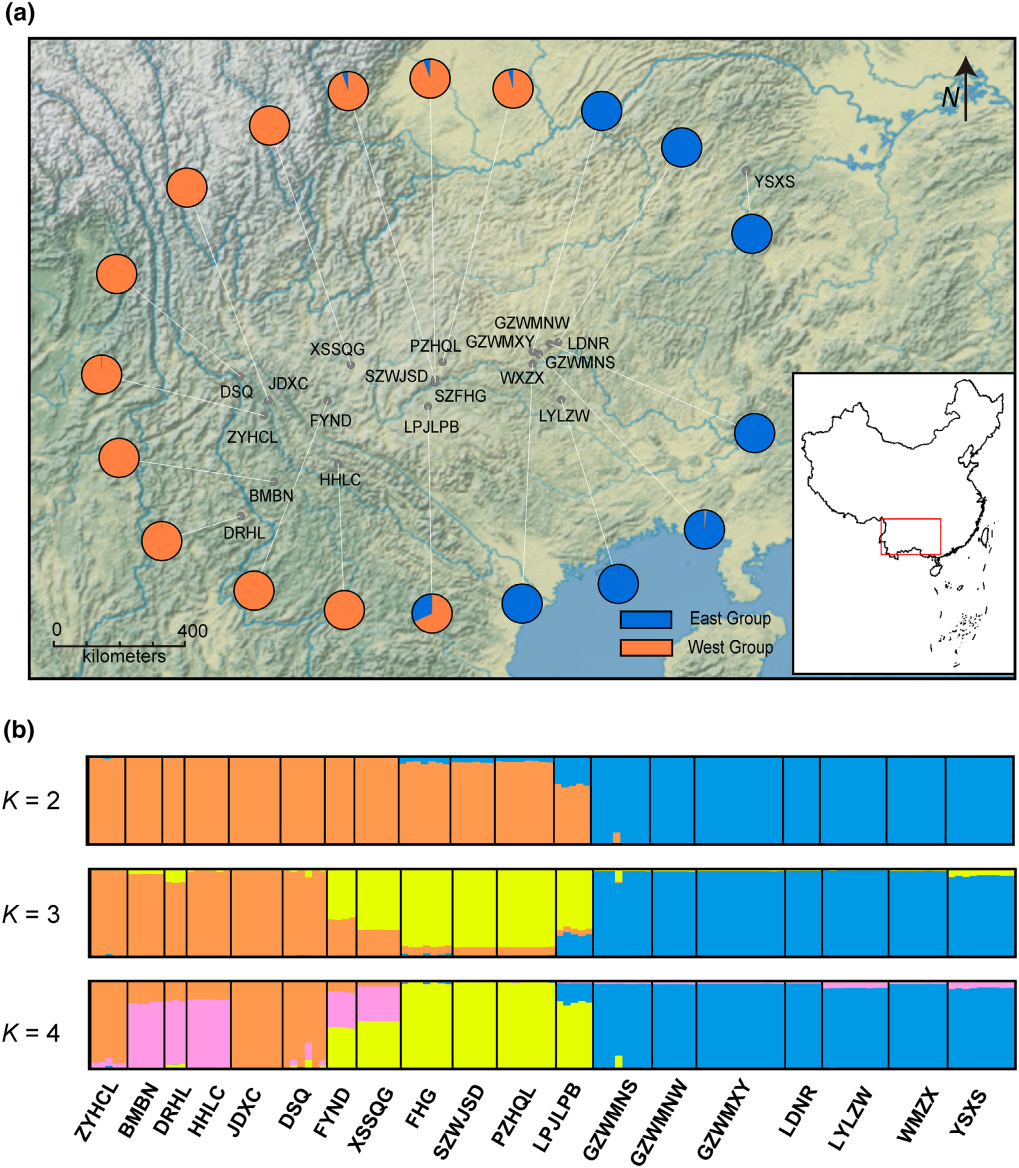
Geographical distribution and population genetic structure of *Amorphophallus yunnanensis*. (a) The sample locations of *A. yunnanensis* populations collected in Southwest China; (b) The STRUCTURE plot of 19 populations (*K* = 2–4).

A population survey and sample collection of *A. yunnanensis* was conducted across southwest China from 2018 to 2020. The sampling nearly covers all potential distribution areas in Southwest China (Yunnan, Guizhou, Guangxi, and Hunan provinces), and leaf samples were randomly selected at intervals of at least 5 m for each population. All 125 individuals were sampled from 19 populations (Table 1 and Figure 2a).

**TABLE 1:** Geographic and genetic characteristics of *Amorphophallus yunnanensis* populations from Southwest China based on SNP data.

	Population	Lon	Lat	Ele (m)	*n*	*PPL*	*H*_O_	*H*_E_	*π*	*F*_IS_
East group					57	0.11072	0.09974	0.12461	0.12587	0.01756
	YSXS	110°13′12.34″	28°46′38.63″	317	9	0.04119	0.11435	0.10413	0.11660	0.00532
	WMZX	106°7′14.62″	24°58′48.79″	432	8	0.04319	0.10354	0.09462	0.10191	−0.00281
	LYLZW	106°40′14.61″	24°16′30.97″	396	9	0.03832	0.08679	0.08559	0.09104	0.01033
	LDNR	106°36′51.53″	25°24′8.31″	436	5	0.01535	0.08727	0.04823	0.05438	−0.05892
	GZWMNW	106°27′8.02″	25°22′25.63″	725	6	0.03757	0.09950	0.08735	0.09581	−0.00598
	GZWMXY	106°7′14.62″	25°13′2.66″	1075	12	0.04834	0.09961	0.10110	0.10586	0.01654
	GZWMNS	106°14′44.26″	25°9′51.25″	726	8	0.04826	0.10644	0.09811	0.10581	0.00048
West group					68	0.14126	0.11761	0.21393	0.21570	0.01967
	LPJLPB	104°6′44.532″	24°8′24.0288″	1300	5	0.04723	0.14673	0.12269	0.13718	−0.0227
	PZHQL	104°23′46.4712″	25°0′52.9164″	1430	8	0.05254	0.12744	0.12374	0.13298	0.01357
	SZWJSD	104°14′56″	24°40′21″	1528	6	0.05131	0.13444	0.12146	0.13323	−0.0024
	SZFHG	104°15′3.6″	24°36′46.8″	1249	7	0.05391	0.13869	0.12659	0.13713	−0.00171
	XSSQG	102°37′50″	24°57′32″	2216	6	0.02355	0.15168	0.07903	0.08674	−0.1189
	FYND	102°11′8″	24°14′53″	1527	4	–	–	–	–	–
	HHLC	102°22′58.66″	23°0′56.29″	1715	6	0.02995	0.08487	0.07532	0.08264	−0.00582
	BMBN	101°9′28.8″	22°39′25.2″	1680	5	0.03376	0.09297	0.08666	0.09732	0.00872
	JDXC	101°2′50.27″	24°15′24.44″	1754	7	0.03773	0.09585	0.09338	0.10093	0.01202
	ZYHCL	100°57′48.13″	23°57′38.25″	1897	5	0.03435	0.08467	0.08032	0.08539	0.00383
	DSQ	100°30′19.02″	24°44′5.19″	2340	6	0.03618	0.11133	0.09764	0.10727	−0.00751
	DRHL	100°30′48″	21°58′55″	1226	3	–	–	–	–	–

Abbreviations: Ele, elevation; *F*_IS_, fixation index; *H*_E_, expected heterozygosity; *H*_O_, observed heterozygosity; Lat, latitude; Long, longitude; *n*, number of individuals; *PPL*, percentage of polymorphic SNP loci; *π*, nucleotide diversity.

### 2.2 Restriction‐site‐associated DNA sequencing

RAD‐seq was performed using established protocols (Baird et al., 2008). In brief, genomic DNA was digested with *EcoR*I endonuclease and then randomly fragmented during the library construction procedure. RAD libraries were paired‐end (PE 150) sequenced by an Illumina NovaSeq 6000 platform. Prior to SNP calling, the genomic data were filtered to remove reads with unidentified nucleotides (Ns) or the proportion of low‐quality bases (Phred value < 5) larger than 50%. We used STACKS v2.54 to assemble the sequencing data and genotype each individual (Rochette et al., 2019). During assembly, the maximum distance allowed between stacks (−M) was set to 3 and the parameter for creating putative alleles (−m) was set to 2. For SNP genotyping, a series of filtering parameter combinations (minimum percentage of individuals in which loci occurred per population (r), minimum number of populations successfully genotyped (p), and minimum percentage of loci presenting across all individuals (R)) were tested to identify the one that maximized SNP numbers and minimized genotyping errors (Table S1). Finally, the parameter set “r75p16R85,” which ensured an average of less than 10% missing genotypes per individual, was selected (Table S1). We only kept the first SNP of each locus to avoid linkage bias. In addition, another dataset with the incorporation of one population of *A. paeoniifolius* was also generated for analyzing gene flow between populations.

### 2.3 Population genetic diversity and differentiation

Population genetic diversity statistics of SNP data were calculated with the “populations” program in STACKS (Rochette et al., 2019), including the percentage of polymorphic SNP loci (*PPL*), observed (*H*_O_) and expected heterozygosity (*H*_E_), nucleotide diversity (*π*), and inbreeding coefficient (*F*_IS_). Populations with less than five individuals (FYND and DRHL) were excluded from all genetic diversity analyses to avoid the bias caused by small population sizes. Population differentiation (*F*_ST_) between each pair of the 19 populations was calculated by the “populations” program, and the significance was tested by Arlequin v3.5 with 1000 permutations (Excoffier & Lischer, 2010; Rochette et al., 2019).

### 2.4 Population structure

We used STRUCTURE v2.3.4 to infer the population differentiation pattern of *A. yunnanensis* (Pritchard et al., 2000). STRUCTURE calculations were conducted with 50,000 burn‐ins and additional 100,000 Markov Chain Monte Carlo (MCMC) chains. The *K* values were set from 1 to 10, with 10 replicates for each *K*. Besides, we calculated the co‐ancestry matrix among all individuals using fineRADstructure (Malinsky et al., 2018). The simulation of fineRADstructure was run by 100,000 burn‐ins and another 100,000 MCMC chains that were sampled every 1000 steps. We also draw principal component analysis (PCA) plots of all individuals with the R package “adegenet” (Jombart, 2008). Finally, a maximum likelihood (ML) tree was constructed using IQ‐TREE v1.6 with 1000 bootstraps (Nguyen et al., 2015).

Analyses of molecular variance (AMOVA) were conducted using Arlequin v3.5 (Excoffier & Lischer, 2010). We assigned populations into genetic groups based on previous genetic structure inference. AMOVA was then conducted to assess the genetic variance among different hierarchical levels (among groups, among populations within groups, and within populations) with 9999 permutations.

### 2.5 Demographic history and gene flow

Stairway Plot v2 was utilized to infer the demographic history of two major lineages of *A. yunnanensis* based on genomic SNPs (Liu & Fu, 2015). The one‐dimensional folded SFS dataset was generated using easySFS (https://github.com/isaacovercast/easySFS). The temporal changes in effective population size (*N*_e_) from 1 × 10^6^ to 1 × 10^3^ years ago were then plotted by Stairway Plot v2. For the lack of a precise mutation rate of *Amorphophallus*, we chose a common mutation rate of 1.0 × 10^8^ per generation (Liu & Hansen, 2017). We set the generation time to 5 years as it usually takes 5 years for *A. yunnanensis* to complete its entire life cycle. Besides, approximate Bayesian computation (ABC) was conducted using DIYABC v2.1 (Cornuet et al., 2014). To reduce the computational time, a subset of 1070 SNPs with <5% missing individuals was used for the analyses. Two genetic groups (west and east groups) were adopted as suggested by genetic structure analyses. We tested two demographic models with permutations. The first model stimulated that the two genetic groups diverged from the common ancestor at the time *t*_d_, and the effect population sizes of the two groups stayed stable. The other scenario proposed that historical population fluctuations have happened in two groups at the time *t*_a_ and *t*_b_, respectively. All parameters were set from 10 to 1000,000, and a total of 2,000,000 permutations were tested with 1000,000 permutations per scenario.

We used the software Treemix v1.13 to test for gene flow between populations of *A. yunnanensis* (Pickrell & Pritchard, 2012). For the analysis, we tested up to five migration events (m) among populations, and one population of *A. paeoniifolius* was used as an “outgroup.” The optimal number of migration edges was determined by the second‐order rate of change across m (Δ*m*) calculated by OptM (Fitak, 2021).

### 2.6 Correlations between genetic, geographic, and environmental factors

We tested isolation by distance (IBD) and isolation by environment (IBE) of *A. yunnanensis*. Geographic distance between populations was calculated from geographic coordinates using GenAlex v6.5 (Peakall & Smouse, 2012). Nineteen climatic variables with a resolution of 2.5 arcminute for each population were downloaded from the WorldClim website (https://www.worldclim.org/; Table S2). To reduce dimensionality, principal components analysis (PCA) for climatic variables was conducted using SPSS Statistics v17.0. The first three PCs explained 92.79% of the total variation (Table S3). Thereby, the data of the first three PCs were used in the follow‐up environmental correlation analysis. The pairwise environmental distances between each population were calculated using the “ecodist” package (Goslee & Urban, 2007). Finally, a series of Mantel tests between genetic distance (*F*_ST_/(1−*F*_ST_)), geographic distance (km) and environmental factors were conducted by GenAlex v6.5 with 1000 replications (Peakall & Smouse, 2012).

### 2.7 Genomic signatures of adaptation

BayPass v2.1 was used to scan the genome of *A. yunnanensis* for signatures of adaptive divergence (Gautier, 2015). First, we calculated the *XtX* statistics with all SNPs. Then, we simulated a pseudo‐observed dataset (POD) of 10,000 SNPs to calibrate the *XtX* statistics. Lastly, we identified outliers based on a 1% threshold of the *XtX* statistics. In addition, we performed genome‐environment association (GEA) analyses using BayPass v2.1, and SNP loci were considered as significantly associated with climatic variables by the 1% threshold of the *XtX* statistics.

To identify putative genes linked to outliers, we extracted candidate genes within 15 kb of the outlier loci from the annotated genome of *Amorphophallus konjac*. Briefly, we aligned the assembled sequence of each outlier locus to the *A. konjac* genome by BLASTN v2.2.28+. Potential functions of genes were annotated with the Gene Ontology (GO), KEGG, SwissProt, and nonredundant protein (Nr) databases.

## 3 RESULTS

### 3.1 Genetic diversity and differentiation

A total of 702.89 Gb of sequencing data was generated from 125 samples of *A. yunnanensis* using RAD‐seq. After filtering, 626.51 Gb of clean data was retained, and the amount of sequencing data per individual ranged from 3.01 to 9.02 Gb, with a mean of 5.01 Gb. The number of sequencing reads per individual ranged from 10,064,414 to 30,061,808. The assembly results showed that the average depth of each sample was 9.92× (Table S4). The resulting dataset consists of 19,034 high‐quality SNP loci with an average missing rate per individual of 8.89% (Table S1). We observed a generally low level of genetic diversity indices across populations, and the highest value of observed (*H*_O_) and expected (*H*_E_) heterozygosity was found in population XSSQG (*H*_O_ = 0.152) and PZHQL (*H*_E_ = 0.127), respectively (Table 1). The percentage of polymorphic SNP loci (*PPL*) was highest in SZFHG (0.054), and the highest nucleotide diversity (*π*) was detected in LPJLPB (0.137). Overall, population LDNR harbored the lowest values in three (*H*_E_, *π*, and *PPL*) out of the four indices (Table 1). A high level of population genetic differentiation with a mean of 0.341 was detected, and pairwise *F*_ST_ between populations ranged from 0.050 (WMZX vs. GZWMXY) to 0.610 (XSSQG vs. LDNR) (Table 2).

**TABLE 2:** Genetic differentiation (pairwise *F*_ST_) values among 19 *Amorphophallus yunnanensis* populations based on genome‐wide SNPs (below diagonal) and significance levels (above diagonal).

	BMBN	DRHL	DSQ	FHG	FYND	GZWMNS	GZWMNW	GZWMXY	HHLC	JDXC	LDNR	LYLZW	SZWJSD	WMZX	XSSQG	ZYHCL	YSXS	LPJLPB	PZHQL
BMBN		NS	*	*	*	*	*	*	*	*	*	*	*	*	*	*	*	*	*
DRHL	0.188		*	*	*	*	*	*	*	*	*	*	*	*	*	*	*	*	*
DSQ	0.286	0.321		*	*	*	*	*	*	*	*	*	*	*	*	*	*	*	*
FHG	0.262	0.258	0.302		*	*	*	NS	*	*	*	*	*	*	*	*	*	*	*
FYND	0.325	0.368	0.352	0.236		*	*	*	*	*	*	*	*	*	*	*	*	*	*
GZWMNS	0.399	0.397	0.422	0.331	0.416		*	*	*	*	*	*	*	*	*	*	*	*	*
GZWMNW	0.443	0.453	0.464	0.355	0.463	0.081		*	*	*	*	*	*	*	*	*	*	*	*
GZWMXY	0.399	0.391	0.426	0.338	0.411	0.050	0.086		*	*	*	*	*	*	*	*	*	*	*
HHLC	0.163	0.259	0.325	0.294	0.364	0.425	0.470	0.424		*	*	*	*	*	*	*	*	*	*
JDXC	0.298	0.336	0.142	0.326	0.371	0.441	0.480	0.445	0.337		*	*	*	*	*	*	*	*	*
LDNR	0.557	0.596	0.570	0.429	0.591	0.138	0.182	0.138	0.590	0.582		*	*	*	*	*	*	*	*
LYLZW	0.433	0.438	0.459	0.360	0.453	0.118	0.145	0.131	0.461	0.477	0.219		*	*	*	*	*	*	*
SZWJSD	0.292	0.291	0.329	0.073	0.269	0.352	0.379	0.356	0.324	0.351	0.462	0.383		*	NS	*	*	NS	*
WMZX	0.418	0.421	0.441	0.343	0.434	0.054	0.092	0.050	0.444	0.458	0.158	0.130	0.366		*	*	*	*	*
XSSQG	0.359	0.380	0.397	0.245	0.294	0.429	0.478	0.428	0.394	0.416	0.610	0.470	0.280	0.449		*	*	*	*
ZYHCL	0.254	0.299	0.146	0.291	0.337	0.410	0.450	0.413	0.298	0.089	0.551	0.445	0.316	0.428	0.378		*	*	*
YSXS	0.478	0.486	0.503	0.396	0.499	0.137	0.162	0.140	0.507	0.519	0.245	0.205	0.419	0.147	0.520	0.486		*	*
LPJLPB	0.339	0.337	0.366	0.175	0.328	0.300	0.334	0.308	0.368	0.389	0.424	0.334	0.196	0.317	0.341	0.354	0.376		*
PZHQL	0.276	0.269	0.316	0.090	0.249	0.343	0.368	0.351	0.307	0.342	0.441	0.371	0.110	0.357	0.256	0.306	0.410	0.199	

Abbreviation: NS, not significant.

* Significance at the 5% nominal level.

### 3.2 Population structure

**FIGURE 3:**
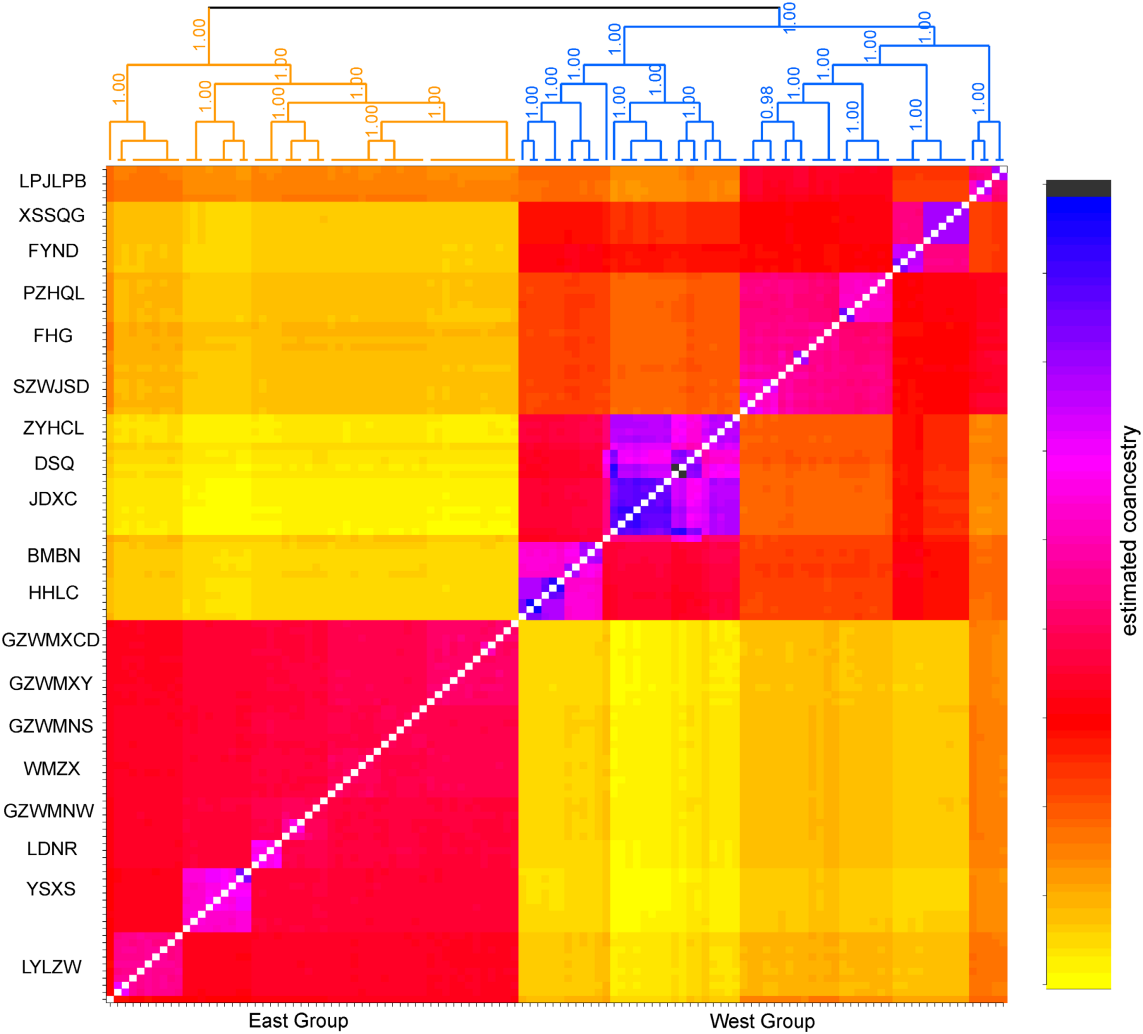
The clustered co‐ancestry matrix of *Amorphophallus yunnanensis* individuals generated by fineRADstructure with genomic SNP data.

**FIGURE 4:**
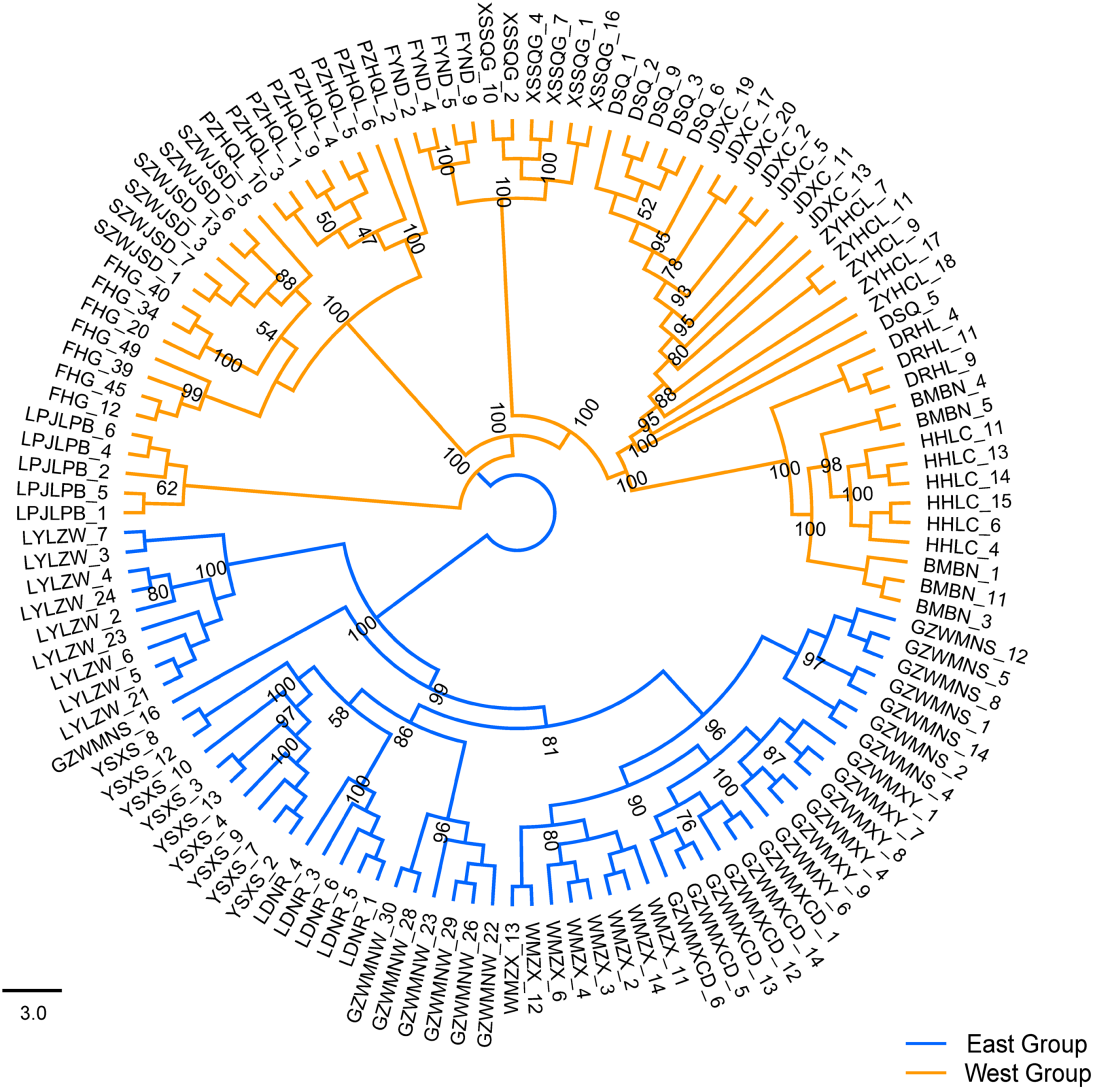
Maximum Likelihood phylogram illustrating genetic relationships among individuals of *Amorphophallus yunnanensis*. Bootstrap values were shown at main nodes.

**FIGURE 5:**
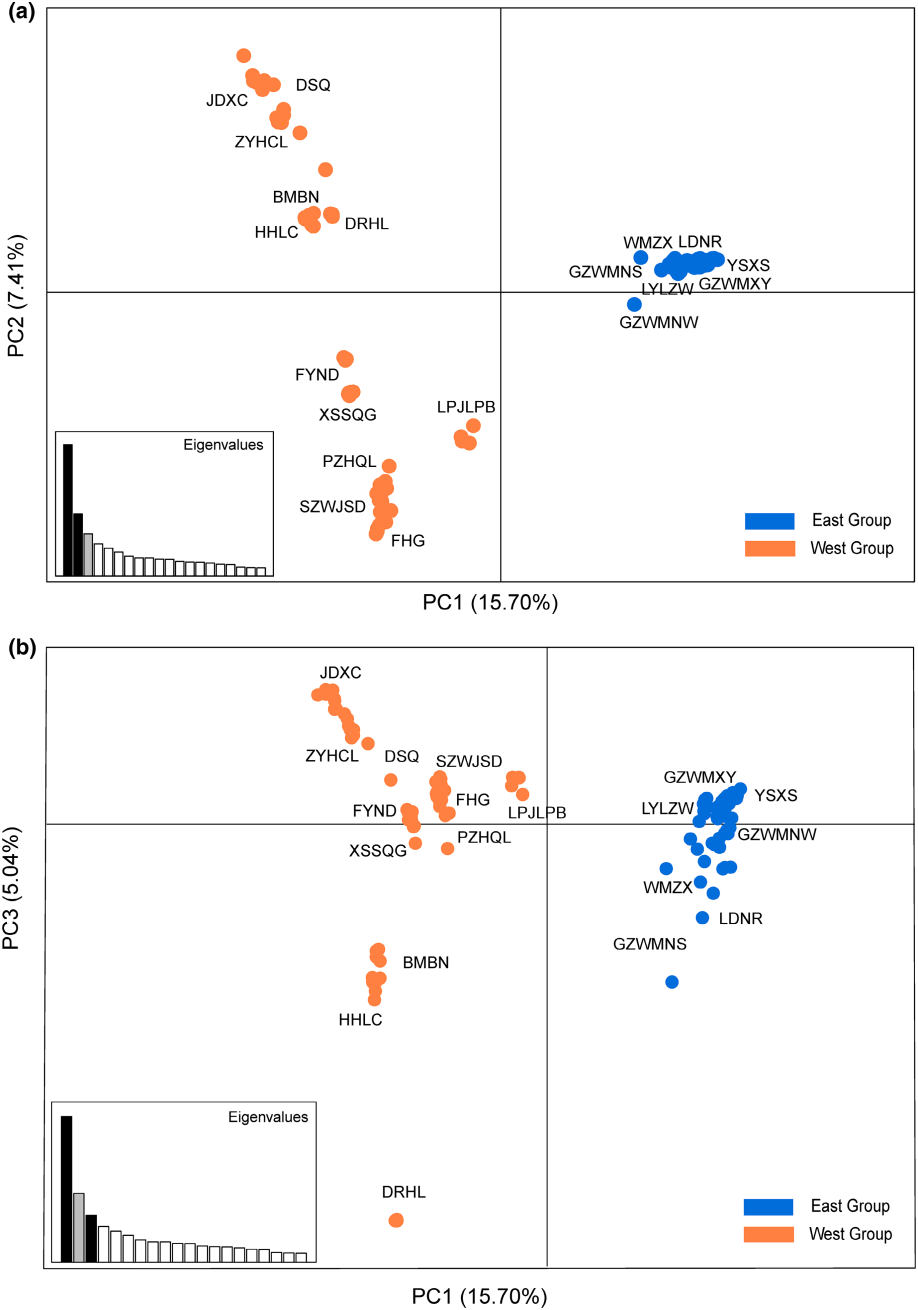
Principal components (PCs) of the variation among *Amorphophallus yunnanensis* individuals with PC1 versus PC2 (a), and PC1 versus PC3 (b).

STRUCTURE analysis based on genomic SNPs detected two distinct genetic clusters (Figure S1). The two clusters were comprised of an east group distributed in the Guizhou plateau and adjacent areas, and a west group mainly in the Yunnan plateau (Figure 2a,b). The 19 sampled populations of *A. yunnanensis* were essentially clustered by geographic proximity, and introgressions were found in four populations located in the adjacent region. Besides, the STRUCTURE results of *K* = 3 and 4 also suggested the genetic uniqueness of populations in the contact region of two plateaus (Figure 2b). The heatmap of fineRADstructure suggested two main genetic groups with a pattern of east–west genetic differentiation. In addition, we observed a subdivision within the west group (Figure 3). The ML phylogenetic tree also identified two clades with a high support value (Figure 4). The PCA analysis pointed out that the first three PCs represented 15.70%, 7.41%, and 5.04% of the total variation, respectively. The PCA plots based on PC1 and PC2 as well as PC1 and PC3 all supported a genetic divergence between east and west groups (Figure 5). The distribution of genetic variance in *A. yunnanensis* was quantified using AMOVA. When assigning populations into two genetic groups, 49.26% of the variation was detected among groups, 19.51% among populations, and 31.23% within populations (all *p*‐value < .001; Table 3).

**TABLE 3:** Results of the analyses of molecular variance (AMOVA) for genetic groups based on genomic SNPs.

Source of variation	df	Sum of squares	Variance components	Percentage of variation	*F*‐statistics	*p*‐Value
Among two groups	1	84188.742	644.807	49.26	*F*_CT_ = 0.493	<.0001
Among populations	17	63305.993	255.361	19.51	*F*_SC_ = 0.384	<.0001
Within populations	231	94442.501	408.842	31.23	*F*_ST_ = 0.688	<.0001

### 3.3 Demographic history

**FIGURE 6:**
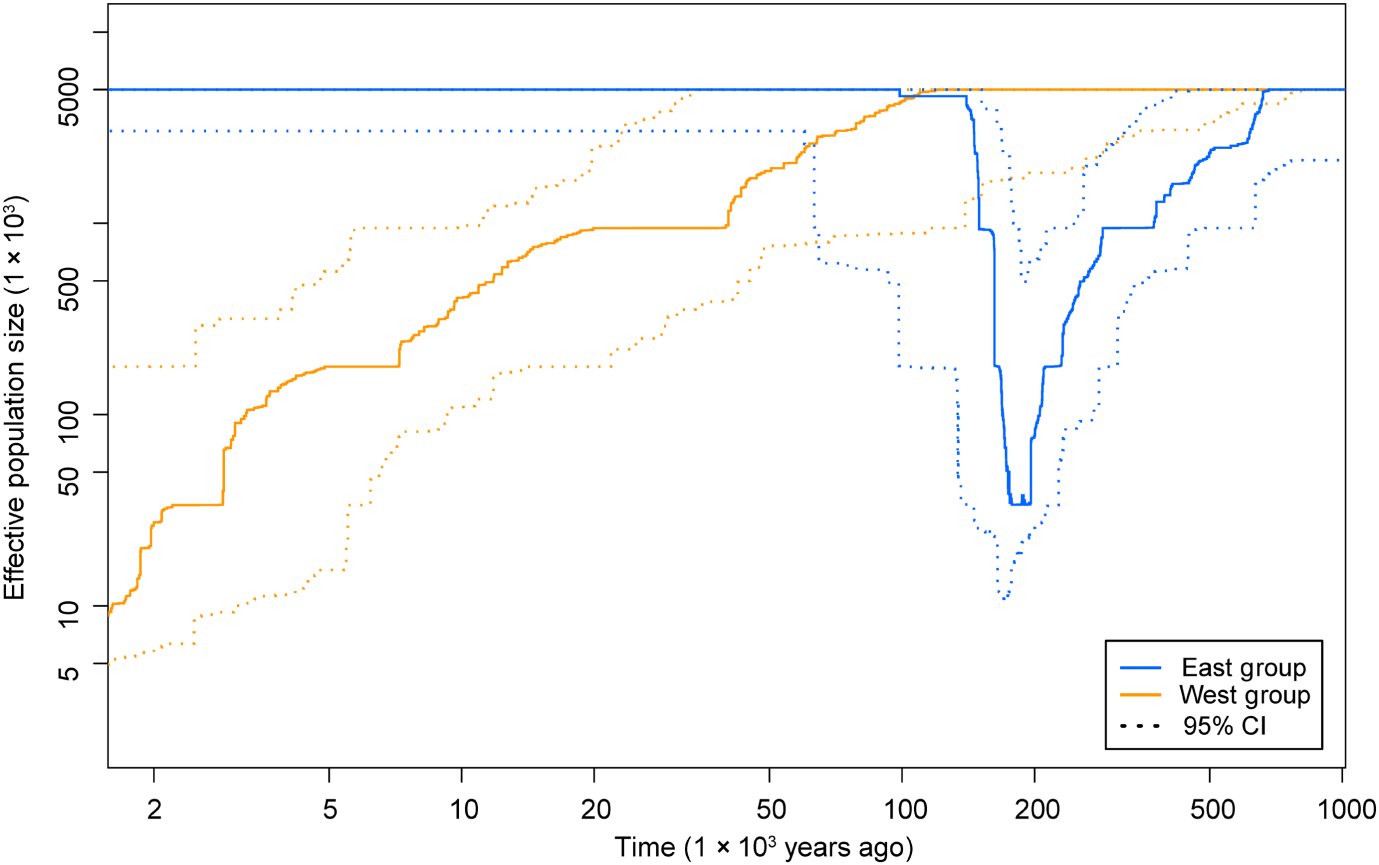
Estimates of the effective population size of *Amorphophallus yunnanensis* through time based on genomic SNPs using stairway plot.

**FIGURE 7:**
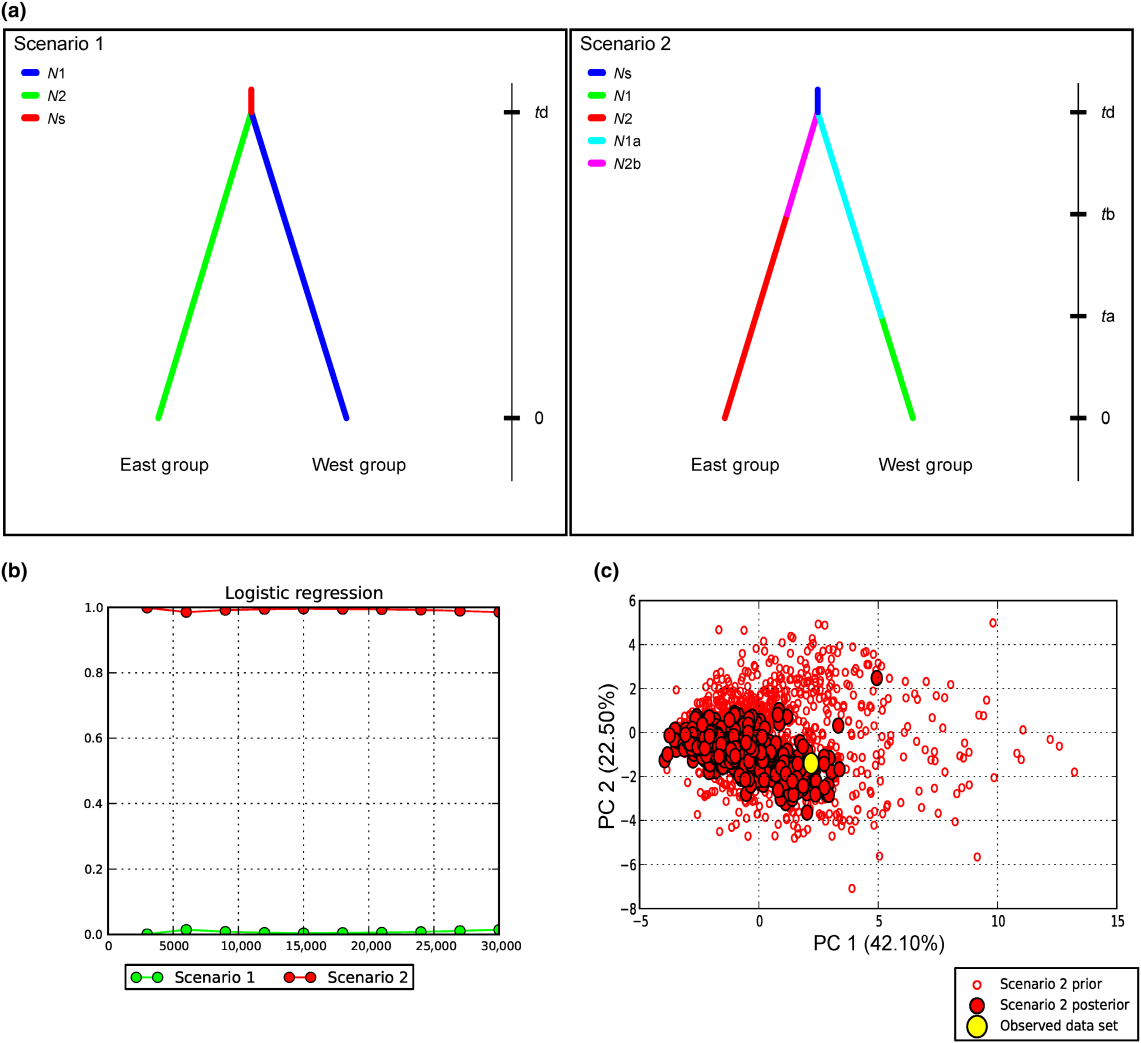
Demographic models based on assumptions in *Amorphophallus yunnanensis* and corresponding posterior probabilities for scenario 2 in DIYABC analysis. (a) Illustrations of two alternative models. Scenario 1 stimulated that the two genetic groups diverged from the common ancestor at the time *t*d, and the effect population sizes stayed stable. Scenario 2 proposed that historical population fluctuations have happened in two groups at the time *t*a and *t*b, respectively. (b) The posterior probability of two models estimated with logistic regression. (c) Model checking and PCA of observed data, comparing prior and posterior distributions of parameters in scenario 2.

**FIGURE 8:**
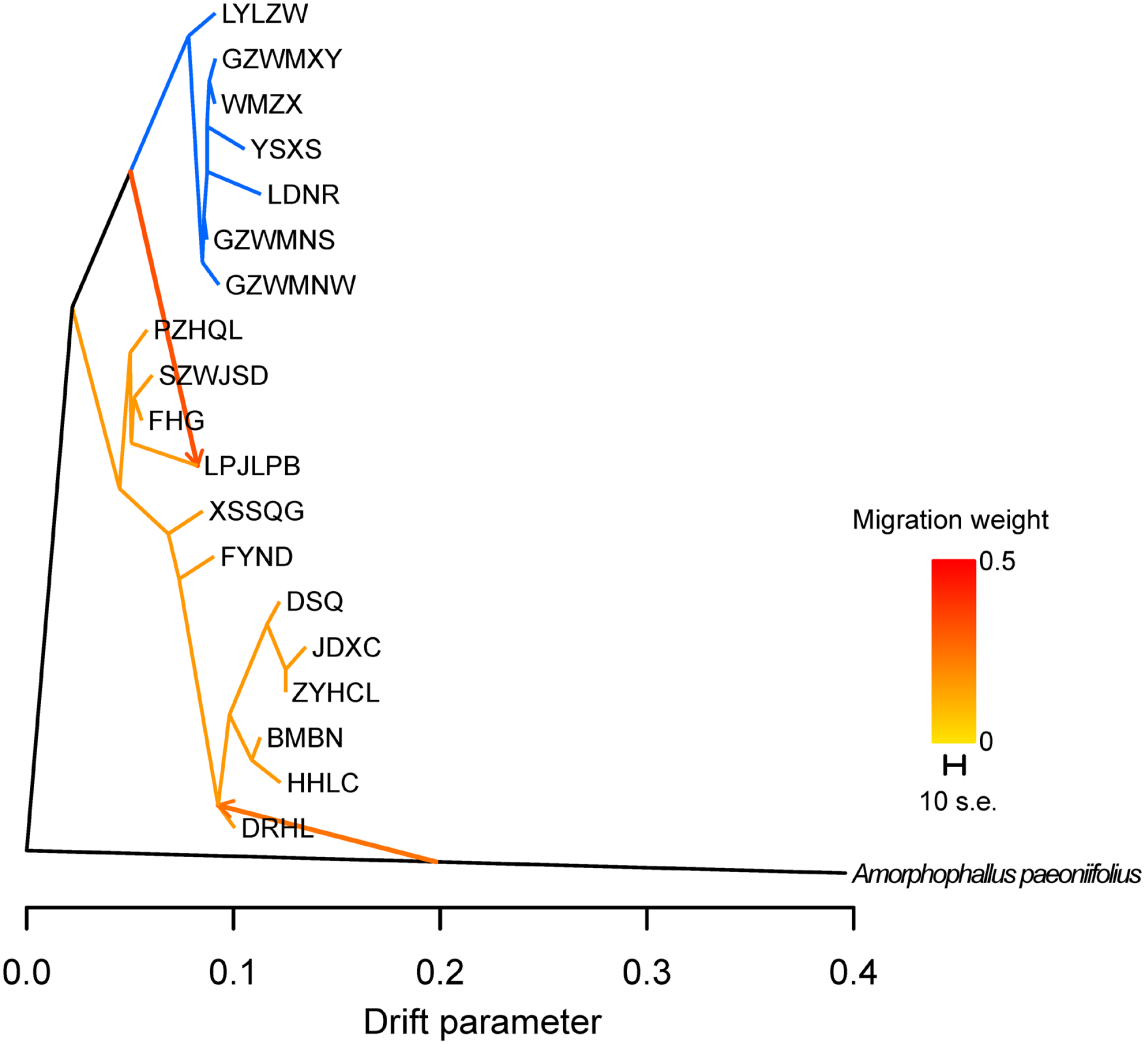
Maximum likelihood tree of *Amorphophallus yunnanensis* populations inferred by Treemix, with the gene flow events depicted with arrows. The branches with blue and orange colors indicate the east and west groups, respectively.

The stairway plot detected that the population size of the west group had been declining ever since 0.1 million years ago (Mya). In addition, the population size of the east group declined at around 0.5 Mya, and then a recent expansion occurred around 0.2 Mya (Figure 6). ABC analyses suggested that scenario 2 was the best‐supported model (Figure 7). The divergence time of the two genetic groups (*t*_d_) was 0.74 Mya, and both groups have undergone historical population declines. Considering a life cycle of 5 years for *A. yunnanensis*, the effective population size of the west group declined at around 0.04 Mya (*t*_a_), and the current population size (*N*_1_) was 10 times smaller than that before the decline (*N*_1a_). Besides, the population size of the east group declined around 0.48 Mya with a reduction of 45% (Table 4). For the analysis of gene flow among populations, two migration events were demonstrated based on the Δ*m* values calculated by OptM (Figure S2). A high level of gene flow was suggested from the common ancestor of the east group to the population LPJLPB, and another weak signal was detected from *A. paeoniifolius* to the west group (Figure 8).

**TABLE 4:** Posterior mean, median, range of 97.5% highest probability distribution (HPD) and mode for eight demographic parameters in scenario 2 for *Amorphophallus yunnanensis* (time is in generations).

	Mean	Median	97.5% HPD	Mode
*N*_1_	7.48 × 10^4^	7.17 × 10^4^	1.37 × 10^5^	6.33 × 10^4^
*N*_1a_	7.45 × 10^5^	7.59 × 10^5^	9.87 × 10^5^	7.49 × 10^5^
*N*_2_	1.83 × 10^5^	1.74 × 10^5^	3.19 × 10^5^	1.72 × 10^5^
*N*_2b_	3.33 × 10^5^	2.61 × 10^5^	9.25 × 10^5^	1.30 × 10^5^
*N*_s_	1.00 × 10^6^	1.00 × 10^6^	1.00 × 10^6^	1.00 × 10^6^
*t*_a_	7.72 × 10^3^	5.28 × 10^3^	2.96 × 10^4^	3.54 × 10^3^
*t*_b_	9.77 × 10^4^	6.85 × 10^4^	3.71 × 10^5^	3.39 × 10^2^
*t*_d_	1.47 × 10^5^	9.06 × 10^4^	6.73 × 10^5^	6.58 × 10^4^

*Note*: *N*_1_ and *N*_2_ are the current effective population sizes of the west and east groups, respectively; *N*_1a_ and *N*_2a_ represent the population sizes of the two groups before population fluctuations; *N*_s_ are the effective population size of the common ancestor of *Amorphophallus yunnanensi*. *t*_a_ and *t*_b_ represent the time of the population fluctuation for the west and east groups, respectively; and *t*_d_ is the divergence time of the east and west groups.

### 3.4 Genomic signatures of adaptation

**FIGURE 9:**
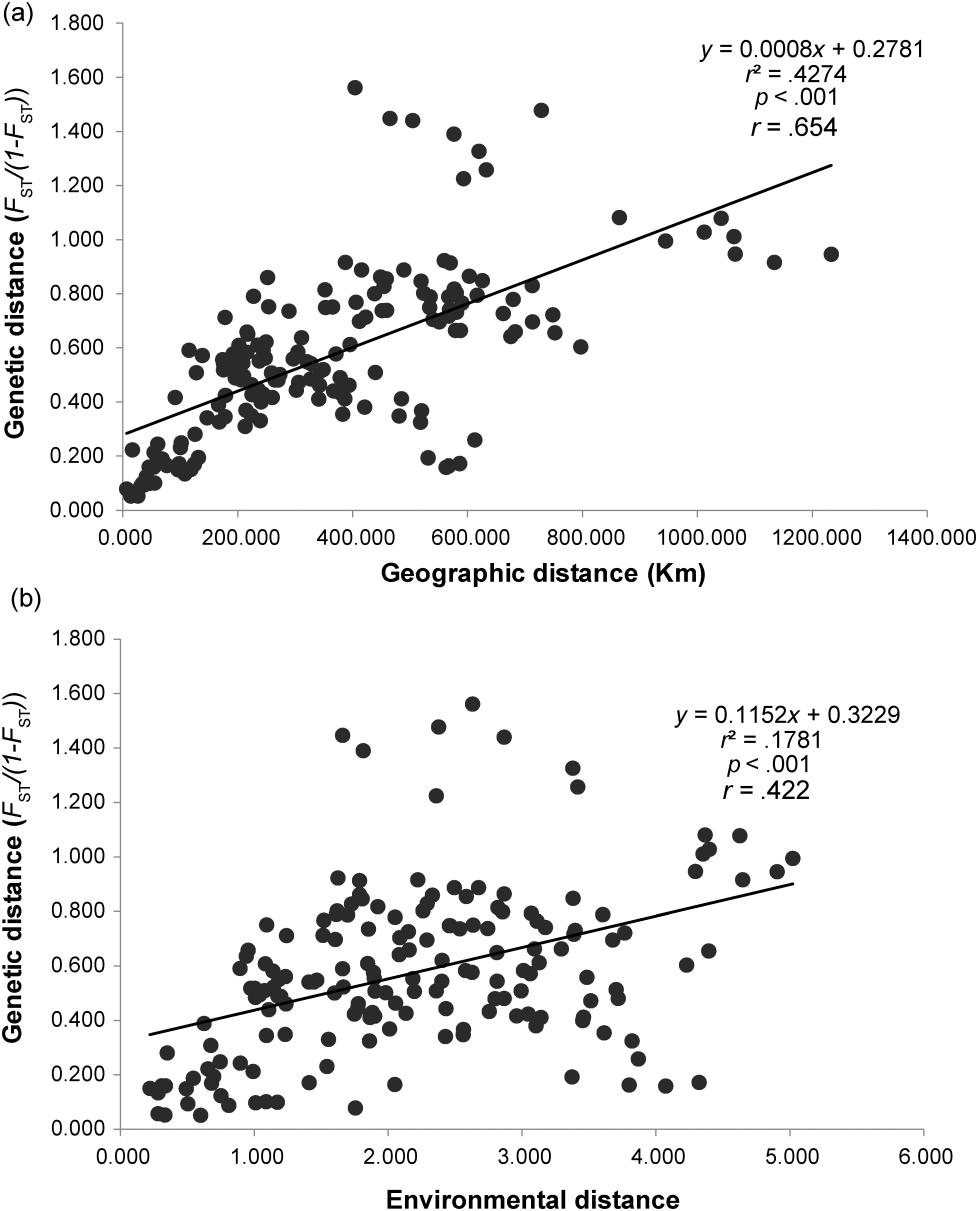
Mantel tests of geographic, environmental, and genetic correlations based on SNP data. (a) Correlation of pairwise geographic distance versus pairwise genetic distance (*F*_ST_/(1−*F*_ST_)); (b) Correlation of pairwise environmental distance versus pairwise genetic distance.

For 19 climatic variables, the first three PCs summarized 42.81%, 28.78%, and 21.77% of the variation, respectively (Table S3). All Mantel tests suggested significant IBD and IBE patterns of *A. yunnanensis* (*p* < .001). However, the correlation between genetic and environmental distances (*r* = .422) was lower than that between genetic and geographic distances (*r* = .654) (Figure 9). For the simulation of BayPass, Spearman's correlation revealed that the posterior estimate of Ω between POD and the real data was 0.998 (Figure S3). This suggested that the simulated data faithfully mimic real datasets and the selected 1% threshold was robust to detect outlier loci. The genome scan based on the *XtX* calibration identified 62 outliers. For the GEA analyses, 46, 39, and 46 loci were found to be significantly associated with Clim_PC1, Clim_PC2, and Clim_PC3, respectively (Table S5 and Figure S4).

The potential gene functions of identified loci were annotated using the *A. konjac* genome. To ensure reliable annotations, we only considered loci with greater than 90% identity and alignment length >200 bp. Twenty‐four out of the 89 loci had BLAST hits to genes, and most of the annotated loci were involved in the molecular function categories, such as nucleotide binding, protein kinase, protein binding, hydrolase, and catalytic activity (Table S6).

## 4 DISCUSSION

### 4.1 Drivers shaping the genetic variation of *A. yunnanensis*

Finding forces that influence the genetic variation of species across the geographical landscape is the ultimate destination of genetic diversity research (Wambulwa et al., 2022). The fixation index expresses the degree of genetic differentiation, and a value of >0.25 is usually considered as a high genetic differentiation level (Wright, 1965). In this study, we detected an average pairwise *F*_ST_ value of 0.341, and most of the *F*_ST_ indices were detected as significant (*p* < .05) (Table 2). Nucleotide diversity for each population (*π*: 0.054–0.137) was lower than statistics calculated from wild populations of *A. paeoniifolius* (*π*: 0.273–0.285) (Gao et al., 2017). These results suggested a pattern of generally low population genetic diversity and high genetic differentiation of *A. yunnanensis*. Similar patterns of genetic variation have also been observed in populations of other *Amorphophallus* species, such as *A. konjac* and *A. albus* (Gao et al., 2018, 2021). *Amorphophallus* are protogynous plant species, and the flowers send forth a strong odor to attract rove beetles as pollinators once pistils are mature (Tang et al., 2020). The limited dispersal ability of these insects might hinder the long‐distance outbreeding of *Amorphophallus* species. Besides, the complicated geomorphological features of the mountainous region of Southwest China have been shown to cause genetic differentiation in many plant species (e.g., Ju et al., 2018; Meng et al., 2015). Mantel test of IBD also suggested that geographic distance accounts for a significant amount of genomic variation in *A. yunnanensis*. Many studies on plants endemic to isolated habitats have suggested that geographical isolation as well as small population size leads to reductions in genetic variation due to genetic drift and limited gene flow (Han et al., 2017). Thus, geographical isolation and genetic drift may be a major driver of the genetic variation in species with fragmented habitats and low dispersal abilities, such as *A. yunnanensis*.

### 4.2 Divergence and secondary contact between east and west groups

Allopatric divergence through geographical isolation is one common mechanism that generates biodiversity in mountain systems (Wiens & Graham, 2005). Our previous study based on SSRs identified three genetic clusters in populations of *A. yunnanensis*. However, this study revealed a clear east–west genetic differentiation pattern, which separated populations distributed in the Guizhou plateau from those in the Yunnan plateau. AMOVA detected a significant amount of variation (49.26%, *p* < .0001) between the two genetic groups, which supported the phylogeographic pattern of *A. yunnanensis*. Several biogeographic boundaries have been proposed for southwest China, which acted as barriers to species dispersal (Ju et al., 2018; Xu et al., 2020). And mountain building has been described as a cause of the genetic differentiation within many plant species in Southwest China (Deng et al., 2020; Wambulwa et al., 2022). In this study, mountains of the Yunnan‐Guizhou Plateau potentially created geographic isolation between populations in these two plateaus. Besides, both the Stairway plot and DIYABC discovered a recent decline in the west group (0.04–0.1 Mya), which coincides with the burst of the last interglacial (LIG). Considering the temperature fluctuations during the Quaternary, Quaternary glacial cycles potentially created niche differences between the Yunnan and Guizhou plateaus (Clift & Webb, 2019; Jia et al., 2020), driving the diversification of *A. yunnanensis* between these two regions.

In addition to two genetic clusters, we also detected genetic admixture in populations in the contact region. Meanwhile, the Stairway plot proposed a very recent population expansion of the east group (around 0.15 Mya), which suggested that range shifts of the east clade caused by Quaternary climatic fluctuations might have led to the secondary contact (Hu et al., 2022; Liu et al., 2012). Treemix also detected gene flow from the east genetic group to populations in the contact region. Finally, a second hybridization caused by demographic expansion from the Guizhou Plateau could have occurred in the recent past, after the initial divergence of two clades, which has been reported in other species in the mountainous region of Southwest China (Ma et al., 2021).

### 4.3 Environmental adaptation

Evaluating the contribution of natural selection is important in studying adaptive evolution (Zhang et al., 2020). Our study revealed a significant pattern of isolation by environment (IBE) in *A. yunnanensis*, which suggested that environmental variables likely contributed to the genetic differentiation except the geographic isolation. Environmental fluctuations caused by mountain formation and isolation might lead to divergent selection (Ren et al., 2017). Outliers identified by GEA could have been targets of divergent selection (Forester et al., 2018; Zhang et al., 2020). BayPass identified highly differentiated SNPs that were significantly associated with climatic factors (Table S5 and Figure S4b–d). Several candidate loci were identified as homologs of critical functional genes, including protein kinases involved in the regulation of signaling pathways, which may have been critical to induce abiotic, stress responses enabling survival in the severe climates in the mountain of Southwest China. For example, loci CLocus_378184 was found within the gene coding mitogen‐activated protein kinase 9 (MAPK 9) (Table S6). MAPK signal transduction cascades are regulators in many aspects of plant biology, such as growth, development, and defense (Andreasson & Ellis, 2010). Although we have not observed consistent morphological differentiations between plants in west and east groups. Considering the *A. yunnanensis*'s sensitivity to sunlight and high temperatures, we speculate that regulation of these pathways would be vital in response to changing altitudes and extreme changes in climate. Besides, genes that participated in the maturation of plants, such as senescence‐specific cystease (SAG39), were found in outlier loci. These genes played an important role in plant growth and development, indicating that morphological adaptations may have been an alternative for *A. yunnanensis* when facing environmental stress (Otegui et al., 2005; Xu et al., 2016).

## 5 CONCLUSION

In summary, we mapped east–west genetic differentiation patterns of *A. yunnanensis* in Southwest China. Our results demonstrated that both the geographic and climatic heterogeneity have promoted and maintained the genetic divergence and demographic dynamics of *A. yunnanensis*. This work has improved our understanding of the drivers of species diversification in the mountainous region of Southwest China. Considering the high morphological heterogeneity of *A. yunnanensis*, further functional studies of the genes that may contribute to the morphological variations will give us more insight into the environmental adaptation of this species.

## AUTHOR CONTRIBUTIONS

**Yong Gao:** Data curation (lead); writing – original draft (lead). **Dongqin Dai:** Investigation (equal); methodology (supporting); writing – review and editing (equal). **Haibo Wang:** Formal analysis (lead); investigation (equal); writing – original draft (supporting). **Weijia Wu:** Investigation (supporting); methodology (supporting). **Penghui Xiao:** Investigation (supporting); methodology (supporting). **Lifang Wu:** Data curation (supporting); investigation (supporting); writing – review and editing (supporting). **Xiaomei Wei:** Data curation (supporting); investigation (supporting); writing – review and editing (supporting). **Si Yin:** Funding acquisition (supporting); project administration (lead); writing – review and editing (lead).

## CONFLICT OF INTEREST STATEMENT

The authors declare no potential conflict of interest.

## Supporting information


Figure S1

Table S1

## Data Availability

Raw sequencing data of RAD‐seq have been deposited in the NCBI database (PRJNA876592). The SNP vcf files of this study are openly available in Figshare at https://doi.org/10.6084/m9.figshare.21057340.
