# Supplementary material for: Genomic insights into differentiation and adaptation of Amorphophallus yunnanensis in the mountainous region of Southwest China
Source: Ecol Evol. 2024 Jan 23;14(1):e10861. doi: 10.1002/ece3.10861 (PMC10805605; doi:10.1002/ece3.10861)
Supplement: Supplementary file 1 — Figure S1 [file ECE3-14-e10861-s002.docx]

**Fig. S1** Δ*K* estimates of the posterior probability distribution for the STRUCTURE analysis based on 19 populations of *Amorphophallus yunnanensis*.


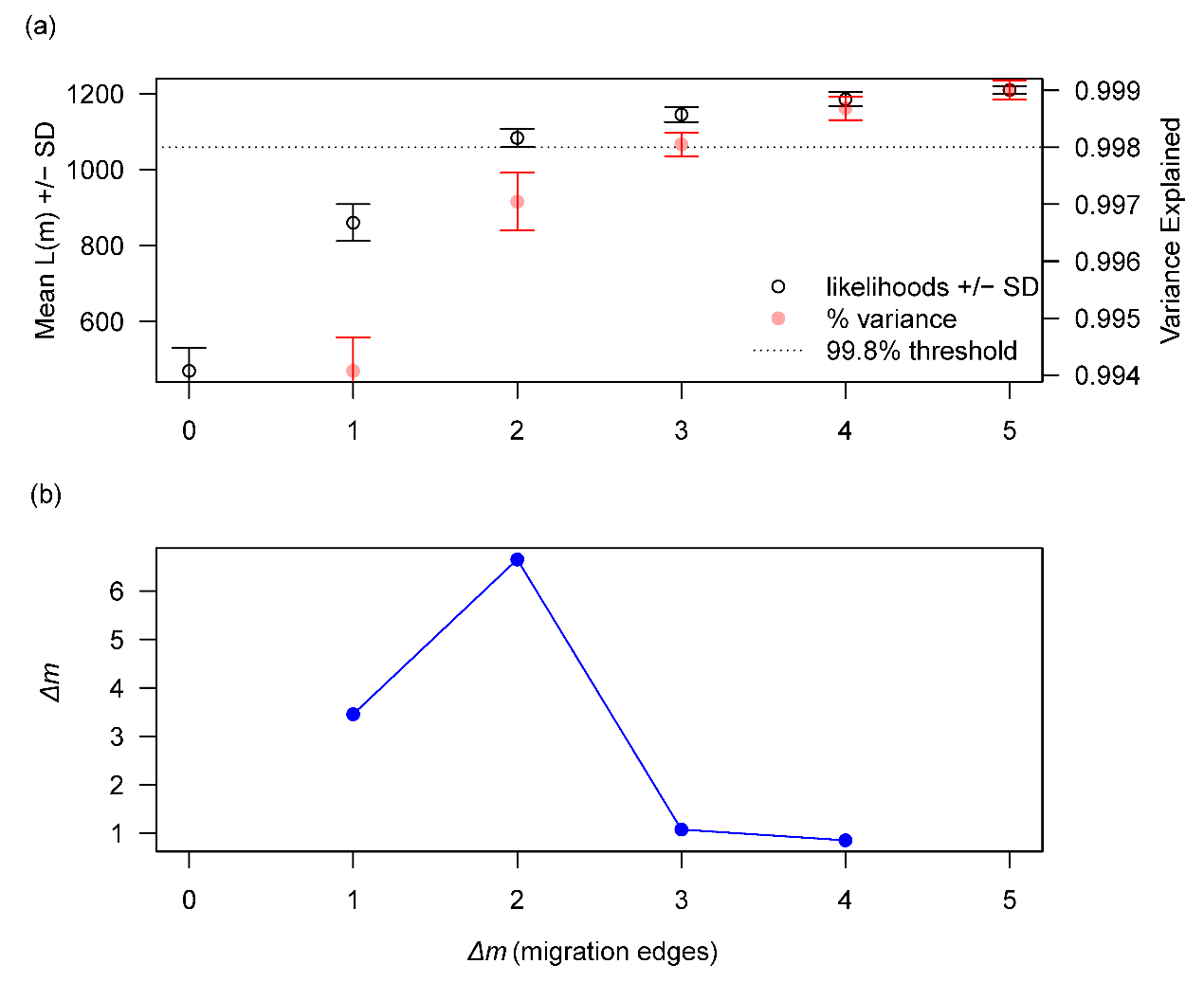


**Fig. S2** The output produced by OptM based on SNP data of *Amorphophallus yunnanensis*. (a) The composite likelihood L(m) (left axis) and proportion of variance explained (right axis) across 5 iterations. The dotted line indicates 99.8% threshold. (b) The second-order rate of change (*Δm*) across values of m.





**Fig. S3** Plot of the Spearman’s correlation between the posterior estimate of Ω obtained with the pseudo-observed data set (POD) and that acquired from the real data set by BayPass.


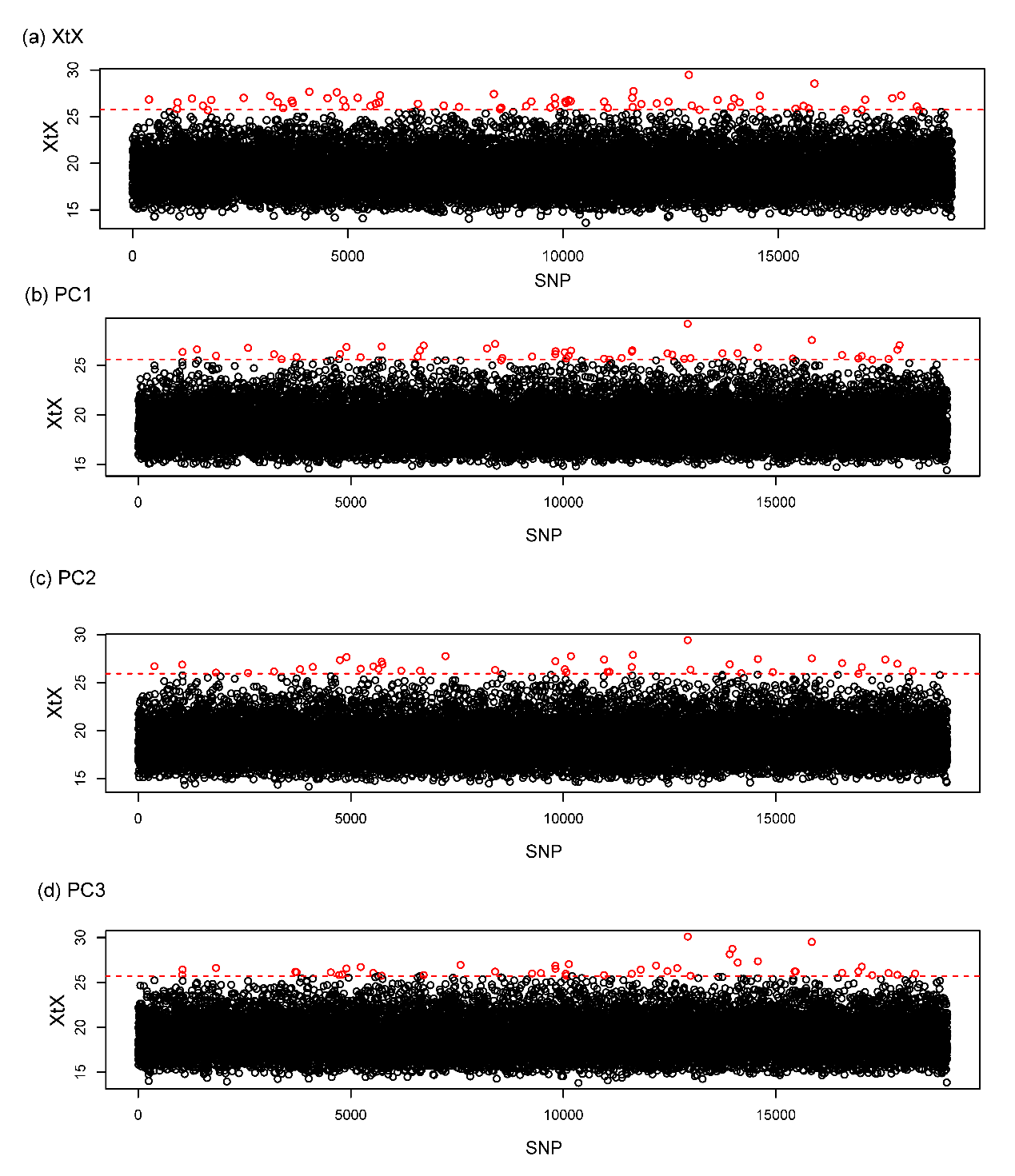


**Fig. S4** The XtX-based outliers detection (a) and environment-SNP association analyses (b, c and d) by the program BayPass. Red dot indicates the significant locus.
